# Epidemiological characteristics of Omicron and Delta SARS-CoV-2 variant infection in Santiago, Chile

**DOI:** 10.3389/fpubh.2022.984433

**Published:** 2022-10-21

**Authors:** Andrea Mella-Torres, Alejandro Escobar, Carlos Barrera-Avalos, Sergio Vargas-Salas, Michelle Pirazzoli, Ulises Gonzalez, Daniel Valdes, Patricio Rojas, Roberto Luraschi, Eva Vallejos-Vidal, Mónica Imarai, Ana María Sandino, Felipe E. Reyes-López, Rodrigo Vera, Claudio Acuña-Castillo

**Affiliations:** ^1^Centro de Biotecnología Acuícola, Facultad de Química y Biología, Universidad de Santiago de Chile, Santiago, Chile; ^2^Laboratorio Biología Celular y Molecular, Instituto de Investigación en Ciencias Odontológicas, Facultad de Odontología, Universidad de Chile, Santiago, Chile; ^3^Hospital de Urgencia Asistencia Pública (HUAP), Santiago, Chile; ^4^Departamento de Biología, Facultad de Química y Biología, Universidad de Santiago de Chile, Santiago, Chile

**Keywords:** Omicron, delta, SARS-CoV-2, pandemic, Chile, epidemiology, vaccination

## Abstract

The variant of concern (VOC) SARS-CoV-2 Omicron (B.1.1529) has been described as a highly contagious variant but less virulent than the current variant being monitored (VBM) Delta (B.1.617.2), causing fewer cases of hospitalizations, symptomatology, and deaths associated with COVID-19 disease. Although the epidemiological comparison of both variants has been previously reported in other countries, no report indicates their behavior and severity of infection in Chile. In this work, we report for the first time the effect of the Omicron and Delta variants in a cohort of 588 patients from the Hospital de Urgencia Asistencia pública (HUAP), a high-complexity health center in Santiago, Chile. This report is framed at the beginning of Chile's third wave of the COVID-19 pandemic, with a marked increase in the Omicron variant and a decrease in the circulating Delta variant. Our results indicated a similar proportion of patients with a complete vaccination schedule for both variants. However, the Delta variant was associated with a higher prevalence of hospitalization and more significant symptomatology associated with respiratory distress. On the other hand, our data suggest that vaccination is less effective in preventing infection by the Omicron variant. This antecedent, with a low severity but high contagiousness, suggests that the Omicron variant could even collapse the primary health care service due to the high demand for health care.

## Introduction

The appearance of the Omicron variant (B.1.1529) was listed as a variant of concern (VOC) by the World Health Organization (WHO) on November 26, 2021 (1). Since then, it has substantially threatened public health and pandemic control through the immune system's evasion and vaccine protection efficacy (2). This variant has a higher affinity for the human angiotensin-converting enzyme 2 (ACE2) compared to the Variant Being Monitored (VBM; classified on April 14, 2022, by the WHO, ceasing to be VOC) Delta (B.1.617.2) (3). Omicron stands out a higher number of mutations in the spike protein (4), which allows a greater infective process and immune evasion. The detection of the Omicron variant co-occurred in several countries in November 2021, with the first case reported in South Africa on November 24, 2021. In Chile, the first documented case of the Omicron variant was identified on November 25, 2021, and was initially considered a variant of public health concern (5). Since then, it took place a sustained and explosive increase in the Omicron variant, doubling the total number of confirmed cases in less than a year and displacing the Delta variant from the 52nd epidemiological week of 2021(December 2021 to January 2022) (5).

Worldwide, the infectivity of the Omicron variant has reached levels higher than those presented in the first wave of SARS-CoV-2, with fewer associated deaths than other variants but more infections per million population (6). While several reports have confirmed the overall lower virulence of this variant compared to Delta (7–9), some waves of infections related to the high spread of Omicron have generated more mortality than reported for the Delta variant, even in the vaccinated population (10). One of the public health policies followed by several countries to counteract the incidence in epidemiological reports of circulating variants is the booster dose vaccination. Several reports indicate that booster doses significantly reduce the symptomatology and severity of disease for the Delta and Omicron cases (11, 12), and increase the immune efficacy compared to the complete vaccination schedule. In Chile, the population vaccinated over the 18 years showed coverage of 93.58% for the first dose, 91.94% for the second dose, and 78.21% for the booster dose (13). CoronaVac (Sinovac Life Science) and BNT162b2 (Pfizer BioNTech) vaccines were administered as first and second doses, while BNT162b2 and ChAdOx1 (Oxford/AstraZeneca) vaccines were administered as booster doses (13). On December 2, 2021, Chile became the country with the highest booster vaccination rate in the world (14).

This study is the first report describing the epidemiological, severity, and symptomatologic characteristics of the VOC Omicron and VBM Delta variants in vaccinated and unvaccinated patients treated at the Hospital de Urgencia Asistencia Pública (HUAP), a high-complexity hospital in Santiago, Chile. Our results indicate a lower prevalence of hospitalization in patients infected with the Omicron variant and a higher prevalence of hospitalization in patients infected with Delta. Symptomatology was similar in both variants; however, shortness of breath and loss of taste and smell were more significant in patients infected with the Delta variant. The behavior of the variants and vaccination is an issue that needs to be addressed in each country and locality because they may behave differently than previously reported in other studies regarding the vaccination schedule and public health policies.

## Materials and methods

### Samples and COVID-19 PCR testing

Nasopharyngeal swab samples (NPSs) were taken from patients who were treated in Hospital de Urgencia Asistencia Pública (HUAP) and treated in other Health centers from Servicio de Salud Metropolitano Central (SSMC) in Santiago de Chile, Metropolitan región.

Samples were analyzed in the Molecular Laboratory of HUAP and the Laboratory of Virology at the Universidad de Santiago de Chile, previously certified by the Chilean Ministry of Health to diagnose SARS-CoV-2 by the RT-qPCR during the pandemic. In HUAP, the SARS-CoV-2 detection was performed using the Allplex™ SARS-CoV-2 Assay kit (Seegene Inc., Seoul, South Korea) according to the manufacturer's instructions. While in Universidad de Santiago de Chile was performed using the ORF1ab gene probe (TaqMan™ 2019nCoV Assay Kit v1 (Thermo Fisher Scientific, part no. A47532) using a one-step strategy as previously reported (15).

### Tracking SARS-CoV-2 variants and waves of infections

The data of variants of SARS-CoV-2 reported by genomic sequencing in Chile were obtained from the open-access platform GISAID after registration and verification (https://www.gisaid.org/). Dataset of RT-qPCR tests and positive cases for the Metropolitan Region of Chile were obtained from the public repository (Ministerio de Ciencia, Tecnología, Conocimiento e Innovación, Government of Chile; https://www.minciencia.gob.cl/covid19/). Data were analyzed by custom software written in Python 3.0.

### Epidemiologic analysis

The epidemiological and demographic analysis was performed using the clinical database provided by the Hospital de Urgencia de Asistencia Pública (HUAP). We analyzed the data generated between November 29, 2021, and January 24, 2022. This includes the first weeks of the third wave of infections registered in Chile, which took place between January and the end of March 2022. Epidemiological and symptomatologic data were obtained from the anamnesis performed in the HUAP Emergency Unit. The information concerning the vaccination status was consulted on the National Immunization Registry (NIR) platform (Ministry of Health; Government of Chile). The databases for the analyses were obtained and stored using the Research Electronic Data Capture (REDCap) software.

### Omicron and Delta genotyping

Viral RNA from samples diagnosed as SARS-CoV-2 positive were genotyped by RT-qPCR. We used the Allplex™ SARS-CoV-2 Variants I (Cat No. RV10286X) and II (Cat No. RV10305X) Assay Kits (Seegene Inc., Seoul, South Korea) according to the manufacturer's instructions. The kit I detects E484K, N501Y, and HV69/70 mutations, while kit II detects L452R, W152C, K417T, and K417N mutations. The combination of the HV69/70, N501Y, and K471N deletion indicates genotyping of the VOC Omicron variant (B.1.1529). In contrast, the combination and detection of L452R and K417N mutations in the NPSs indicate the presence of the VBM Delta variant (B.1.617.2).

### Statistical analysis

Chi-square analysis was performed to analyze the epidemiological, clinical and vaccination characteristics of the patients studied. Z-test was performed for symptom analysis. A *p* < 0.05 was considered statistically significant. The analyzes were performed using GraphPad Prism 8.0.2 software.

### Ethics statement

This study was authorized by the Ethical Committee of the University of Santiago of Chile (No. 226/2021) and the Scientific Ethical Committee of the Central Metropolitan Health Service, Ministry of Health, Government of Chile (No. 79/2022), and following the Chilean law in force.

## Results

### Prevalence of hospitalization in patients performed with the Omicron and Delta variant

**Figure 1:**
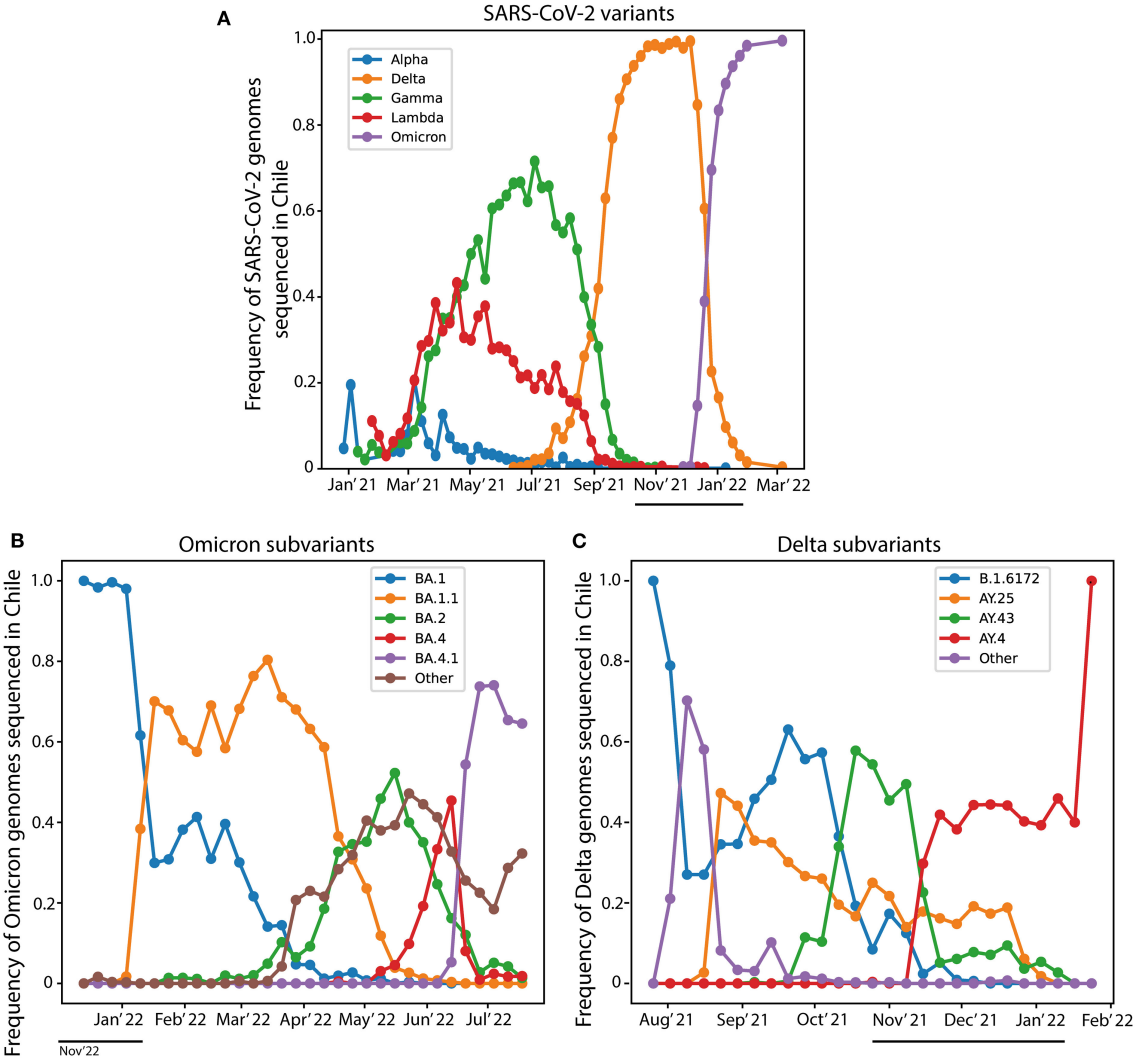
Prevalence of SARS-CoV-2 variants in Chile. The frequency of SARS-CoV-2 genomes identified in Chile was obtained from the GISAID webpage. **(A)** The circulation frequency of the main variants of SARS-CoV-2 genomes data sequenced in Chile. **(B)** The circulation frequency of the Omicron subvariants sequenced in Chile. **(C)** The circulation frequency of the Delta subvariants sequenced in Chile. The black line shows the study period from November 2021 to January 2022. The data for the Omicron variant is available only since January 2022 in the GISAID webpage.

**Figure 2:**
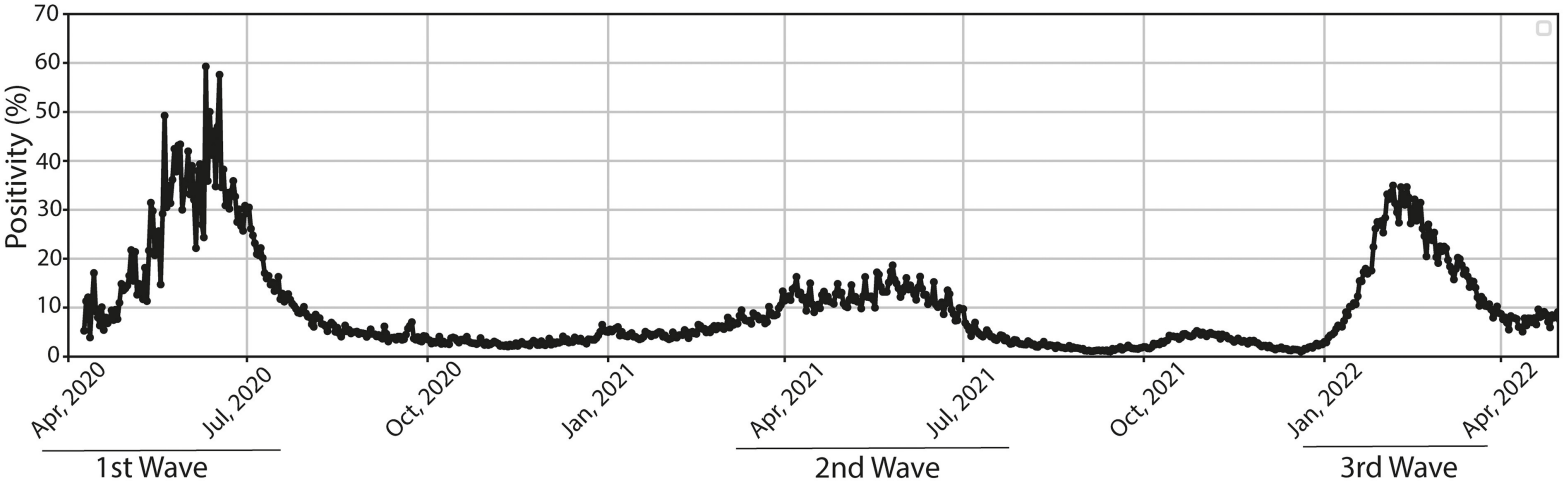
Positivity of COVID-19 in the Metropolitan Region of Santiago. The positivity (%) in the Metropolitan region is shown from April 2020 to April 2022. The first wave is indicated between the months of April and July 2020, the second wave corresponds between March 2021 and the end of July of the same year, while the third wave of infections begins between the months of January 2022 and April 2022.

We analyzed the prevalence of the variant of concern (VOC) Omicron and the variant being monitored (VBM) Delta from June 2021 to March 2022. For this purpose, we used the SARS-CoV-2 genomic sequences information reported for Chile in the Global initiative on sharing all influenza data (GISAID) (16). The progression over time of the most relevant SARS-CoV-2 variants is showed in Figure 1A. The Delta variant began to decrease steadily in the Chilean population in mid-December 2021. Remarkably, the Omicron variant increased in the same trend as the Delta variant, although in the opposite direction. In the study period (November 2021 to January 2022), the main Omicron subvariants circulating in Chile were BA.1 and BA.1.1 (Figure 1B). At the same time, the main subvariants of Delta corresponded to AY.43, AY.4, and AY.25 (Figure 1C). Genotyping of SARS-CoV-2 variants only indicated the presence of Omicron or Delta without specifying the subvariant involved in each infection. This data indicates that, at the epidemiological level, the Delta variant was displaced by the Omicron variant. Moreover, the Omicron variant has been present in most infected populations since November 2021 (Figure 1). This antecedent acquires particular relevance when we observe the whole progression of the pandemic. In this context, the third wave was characterized by an increase in positivity from January 2022, whose infection peak was observed in February 2022 (Figure 2). Our data show that this peak in the third wave of pandemic infection coincides with the positioning of the Omicron variant as the clearly predominant and even almost exclusive because the no register of other main circulating variants at that time.

We evaluated the epidemiological characteristics induced by Omicron and Delta variants at this time. For this purpose, we analyzed a cohort of patients obtained from the Hospital de Urgencia de Asistencia Publica (HUAP), a highly complex health center in Santiago de Chile, between weeks 48 of 2021 (November 29, 2021) until week 4 of 2022 (January 24, 2022). The 3,162 diagnostic tests from Nasopharyngeal Swab Samples (NPSs) showed positivity of 22.3% (705 positive cases). Then, 588 NPSs were genotyped because they passed the criteria of a quantification cycle (Cq) lower or equal to 35. The results determined that 534 NPSs (90.5%) corresponded to the Omicron (B.1.1529) variant, while 54 (9.1%) samples to Delta (B.1.617.2) variant. On the other hand, one sample was identified as Alpha (B.1.1.7) and Gamma (P.1) variants (0.2%, respectively) (Supplementary Figure 1).

The median age distribution of infected patients was similar for both variants. The cases reported in patients infected with the Omicron variant were significantly higher in women than in men. In contrast, the male gender presented a higher prevalence of infection with the Delta variant (*X*^2^ = 3.920, *p* < 0.05) (Table 1). We then analyzed the number of hospitalizations as a critical factor in determining the severity of infection of the variants analyzed. We determined that 5.2% (32 patients) were hospitalized for COVID-19, registering 4.6% and 14.8% of patients infected with the Omicron and Delta variants, respectively (*X*^2^ = 5.92, *p* < 0.01; Table 1). Although patients infected with the Delta variant represented less than those infected with Omicron, the relationship and proportionality indicate that patients infected with Delta are 3.4 times more likely to need to be hospitalized (with oxygen requirement) in healthcare centers (Table 1). Only one death was reported from the total cohort analyzed, corresponding to a patient infected with Omicron, representing a 0.2% death associated with this variant, compared to 0% reported for Delta (Table 1). Accordingly, the lack of mortality data makes it possible to perform a comparative analysis between patients infected with Omicron and Delta variants. The data suggest that the Omicron variant causes less severe COVID-19 than the Delta variant (according to the analysis of hospitalizations and oxygen requirements).

**Table 1:** Epidemiological characteristics of patients infected with the Omicron and Delta variant from a cohort of patients belonging to the Hospital de Urgencia Asistencia Pública (HUAP), Santiago, Chile.

	***N*°(%)**	**Omicron**	**Delta**	**Significance, *p*-value, *X*^2^**
Total patients	588	534	54	n/a
Age median (IQR) [range]		34 (28–48) (15–99)	35 (29–52) (19–95)	n/a
**Gender, N°(%)**
Female	331 (55.9%)	306 (57.1%)	23 (42.6%)	*, 0.0239, 3,920
Male	261 (44.1%)	230 (42.9%)	31 (57.4%)	
**Hospitalized patients**
Not hospitalized (Ambulatory patients without oxygen)	513 (86.7%)	469 (87.5)	44 (79.6%)	**, 0.0075, 5.920
Patients hospitalized by COVID-19 (oxygen requirement)	32 (5.4%)	23 (4.3%)	8 (14.8%)	
Mortality	1 (0.2%)	1 (0.2%)	0 (0%)	n/a
**N° Symptoms per patient**
Median (IQR) [range]	4 (2–5) (1–9)	4 (2–5) (1–9)	4 (2–5) (1–9)	n/a

*p < 0.05; **p < 0.01; n/a, not apply.

### Effect of vaccination on contagion by the Omicron and Delta variant

It is striking that vaccination does not seem to be a relevant factor in such cases. Both groups had similar vaccination percentages with a complete schedule of two doses (23.8% and 25.9% for Omicron and Delta cases, respectively). Almost 50% of those infected with the Omicron variant received a booster dose, while 33% of cases infected with Delta received it. We found no differences between the effect of two doses and one dose on the prevalence of infection by the Omicron or Delta variant. We also found no differences between the effect of a booster dose after a complete vaccination schedule (two doses) on the prevalence of infection by one variant or another (Table 2). However, our analysis indicated that vaccination helps reduce the spread of the Delta variant in the Chilean population. On the other hand, our data suggest that vaccination is less effective in preventing infection by Omicron because of the total number of patients infected by Omicron (86% of the reported cases vaccinated with at least one dose) (Table 2). These data suggest that vaccination has low effectiveness in preventing Omicron from spreading.

**Table 2:** Effect of each vaccination dose on the prevalence of Omicron and Delta variant infection in a cohort from Santiago of Chile.

	**Total (%)**	**Omicron cases**	**Delta cases**	**Significance, *p*-value, *X*^2^**
**COVID-19 vaccination**
Yes	497 (84.0)	461 (86.0)	36 (66.7)	**, 0.0018, 9.344
No	95 (16.0)	75 (14.0)	18 (33.3)	
Two-dose and Booster dose	311 (52.5)	293 (49.5)	18 (33.3)	n/s, 0.0938, 1.737
Two-dose	155 (26.2)	141 (23.8)	14 (25.9)	
Two-dose	155 (26.2)	141 (23.8)	14 (25.9)	n/s, 0.346, 0.1559
One-dose	31 (5.2)	27 (4.6)	4 (7.4)	

The number of positive nasopharyngeal swab samples and its percentage is detailed for each column.

n/s, not significant; **p < 0.01.

The distribution of the symptoms generated by the Omicron and Delta variants was similar in almost all the symptoms. However, in patients infected with the Delta variant, there was a significant increase in respiratory distress (dyspnea) and loss of taste and smell (Anosmia/Ageusia), with a prevalence of approximately 10% more than the symptoms generated by the infection (Table 3). We hypothesize that the higher prevalence of hospitalizations for patients with Delta variant could be related to a most significant symptomatology of dyspnea.

**Table 3:** Symptomatologic characteristics associated with patients infected by Omicron and Delta variant in a cohort from Santiago of Chile.

	**Omicron cases**	**Delta cases**	**Significance, *p*-value**
Symtoms	*N*° (%)	*N*° (%)	
Fever	265 (49.4)	28 (51.9)	n/s
Cough	297 (55.4)	31 (57.4)	n/s
Fatigue	188 (35.1)	18 (33.3)	n/s
Anosmia/Ageusia	30 (5.6)	7 (13.0)	*
Nasal congestion	142 (26.5)	11 (20.4)	n/s
Throat pain	19 (3.5)	1 (1.9)	n/s
Headache	235 (43.8)	24 (44.4)	n/s
Myalgias	315 (58.8)	32 (59.3)	n/s
Odynophagia	228 (42.5)	18 (33.3)	n/s
Nausea/Vomiting	69 (12.9)	9 (16.7)	n/s
Diarrhea	56 (10.4)	6 (11.1)	n/s
Chills/Vertigo	25 (4.7)	3 (5.6)	n/s
Dyspnea	47 (8.8)	11 (20.4)	**
Loss of appetite	6 (1.1)	2 (3.7)	n/s
Confusion	4 (0.7)	0 (0.0)	n/s
Chest pain	22 (4.1)	1 (1.9)	n/s
Anxiety	1 (0.2)	0 (0.0)	n/s
No symptoms	101 (18.8)	5 (9.3)	n/s
Total	**536**	**54**	

The number of positive nasopharyngeal swab samples and its percentage is detailed for each column.

*p < 0.05; **p < 0.01; n/s, not significant.

## Discussion

Previous studies have reported comparative epidemiological studies on the severity and virulence-associated with infection by SARS-CoV-2 Omicron and Delta variants. For example, Bouzid et al. (17), indicated that the Omicron variant infection was associated with a lower risk of admission to the intensive care unit (ICU) and death compared to the Delta variant, which induced more significant respiratory problems and 11% more cases of hospitalizations in patients residing in the Metropolitan area of Paris, France. Another analysis conducted in UK concluded that Omicron infections were less severe than those caused by Delta, even in unvaccinated patients. However, severity was directly proportional to age, and vaccination was less effective in preventing Omicron infections (11). This same effect has been reported even in pediatric patients under 5 years of age (18). By contrast, some studies in the USA have reported the opposite effect, where the Omicron variant causes more mortality (10). Sometimes suggesting a substantial increase in mortality even for a variant classified as less virulent. However, more studies confirm the low severity of Omicron compared to Delta, because of less involvement of the lower respiratory tract. This low virulence of Omicron has led countries to reconsider the current public health policies for fewer health restrictions on the population (19). Despite all the reproduced epidemiological reports on the virulence of both variants of SARS-CoV-2 in infected patients from different countries, with different vaccination strategies and population genetic backgrounds, no report describes the same situation in the Metropolitan Region of Santiago de Chile.

In this work, we compare the epidemiological characteristics associated with the COVID-19 disease generated by the Omicron and the Delta variant in a cohort of the Public Assistance Emergency Hospital (HUAP), a high-complexity health center in Chile. Our results indicate that the Omicron variant generally results in less severe disease, with less risk of hospitalization than that caused by the Delta variant. These data were associated with symptoms of respiratory distress and loss of smell and taste in more than an 10% of the cases infected with Delta variant. In terms of immunization, the cohort presented 86% and 66% with at least one dose of vaccine in those infected with Omicron and Delta, respectively. This suggests the low effectiveness of vaccines to prevent infections by Omicron. According to data from the Ministry of Health, since January 2022, this variant has become the predominant one in Chile, representing 100% of the circulating virus to date (5).

Current information allows us to determine that the Omicron variant has a lower infective capacity in the lower respiratory tract (20). These disease characteristics induce less damage to the airways and lungs. Omicron infection generally allows outpatient clinical management, unlike previous variants such as Gamma and Delta, where hospitalization and higher mortality were characteristics of high lower respiratory and pulmonary compromise (21, 22). Our clinical data extends those presented by Maisa et al. (23) and obtained in France for the first cases of infection by this variant. The authors indicate a mild clinical representation of the manifestation by Omicron and constant genomic surveillance of the country. Similar to South Africa, the data generated suggested a reduction in disease severity in adults with Omicron infections (24). Compared to the Delta variant, cohort reports generated in Norway, Canada, Germany, and the UK also described the same results with reduced hospitalization and deaths (9, 11, 25, 26). Therefore, and it has been determined in other countries, the Omicron variant has shown high contagiousness, demonstrated by increases in the disease rate well above those defined in different waves. Our findings were consistent with the reports of the different countries. In Chile, at the end of January 2022, 93.4% of confirmed positive cases were associated with the Omicron variant (5). The protection provided by vaccination could play a fundamental role in the lower severity induced by the variants. In this way, a global comparison between the different vaccine protocols and manufacturer strategies against Omicron and Delta infection would be interesting, especially considering that hospitalization and mortality from the Delta variant occurred in patients without a vaccine. Currently, the mortality rate in Chile is 0.28 and 0.63 for patients vaccinated with booster doses, compared to patients without vaccines or incomplete vaccination, respectively, until June 2022 (27). The data are in accordance with those reported in a study from China (28).

On the other hand, although our work is consistent with that previously reported by other countries, the low number of patients infected with Delta in the analyzed cohort did not allow a comparison between age groups. This analysis seems essential because, although the Omicron variant is less virulent, it can cause severe manifestations in elderly patients or patients with previous comorbidities (29). In addition, this Our study does not differentiate between types of vaccines, which would be interesting to analyze in future studies to determine the best vaccination protocol for future booster doses or public health policies. Finally, our study indicates that the Omicron variant generates less severity than the Delta variant. However, it is necessary to maintain genomic surveillance of the population because the high contagiousness of Omicron even in vaccinated patients could generate new variants of SARS- CoV-2 that could once again compromise public health.

## Data availability statement

The original contributions presented in the study are included in the article/Supplementary material, further inquiries can be directed to the corresponding author/s.

## Ethics statement

This study was authorized by the Ethical Committee of the University of Santiago of Chile (No. 226/2021) and the Scientific Ethical Committee of the Central Metropolitan Health Service, Ministry of Health, Government of Chile (No. 79/2022), and following the Chilean law in force.

## Author contributions

CA-C, RV, and FER-L: conceptualization. CA-C, SV-S, and RV: methodology. AMS, CA-C, and MI: validation. PR: formal analysis. AM-T, RL, RV, MP, EV-V, and UG: investigation. CA-C, MI, FER-L, and AMS: resources. CA-C and PR: data curation. CA-C, AE, and AM-T: writing—original draft preparation. CB-A and FER-L: writing—review and editing. DV: visualization. CA-C, FER-L, and AMS: supervision. FER-L and AMS: project administration and funding acquisition. All authors have read and agreed to the published version of the manuscript.

## Funding

The Laboratory of Virology had the support from the COVID-19 diagnosis in the University laboratories network (Ministry of Sciences, Ministry of Health, Government of Chile) for diagnosis tasks. The authors also thank to the rapid assignment of resources for research projects on the Coronavirus pandemic (COVID-19) (project number COVID1038; Agencia Nacional de Investigación y Desarrollo de Chile (ANID), Government of Chile), Fondecyt regular project numbers 1201664 (MI) and 1211841 (FER-L), and Fondecyt iniciacion project number 11221308 (EV-V) (ANID, Government of Chile). We also thank to the DICYT-USACH project number 021943AC (CA-C). The funders had no role in study design, data collection, and analysis, decision to publish, or preparation of the manuscript.

## Conflict of interest

The authors declare that the research was conducted in the absence of any commercial or financial relationships that could be construed as a potential conflict of interest.

## Publisher's note

All claims expressed in this article are solely those of the authors and do not necessarily represent those of their affiliated organizations, or those of the publisher, the editors and the reviewers. Any product that may be evaluated in this article, or claim that may be made by its manufacturer, is not guaranteed or endorsed by the publisher.
