# Supplementary material for: Epidemiological characteristics of Omicron and Delta SARS-CoV-2 variant infection in Santiago, Chile
Source: Front Public Health. 2022 Oct 21;10:984433. doi: 10.3389/fpubh.2022.984433 (PMC9634544; doi:10.3389/fpubh.2022.984433)
Supplement: Supplementary file 1 [file Image_1.pdf]

**SUPPLEMENTARY MATERIAL**

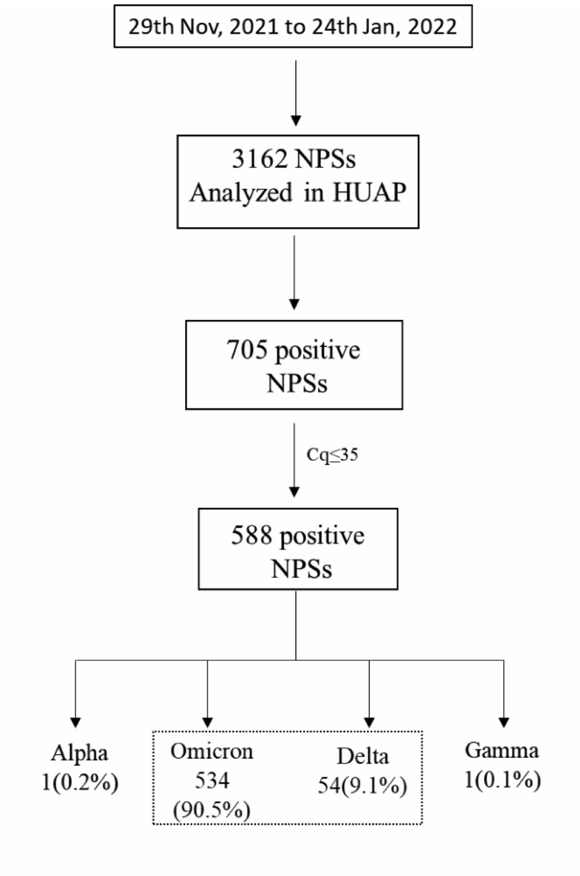


**Supplementary Figure 1. Diagram of patients selected for the study cohort**. Clinical data of positive patients for COVID-19 were obtained from November 29, 2021, to January 24, 2022, from the Public Assistance Emergency Hospital (HUAP), Santiago, Chile. In this period of time, nasopharyngeal swab samples (NPSs; n= 3,162 samples) were analyzed at HUAP, of which 705 were positive for COVID-19. The samples with an amplification value of Cq<35 were genotyped by RT-qPCR, obtaining one sample corresponding to the Alpha variant, 532 NPSs identified for the Omicron variant, 54 NPSs for Deltavariant, and one sample corresponding to the Gamma variant.
